# Fermented‐*Rhus verniciflua* extract ameliorate *Helicobacter pylori* eradication rate and gastritis

**DOI:** 10.1002/fsn3.2055

**Published:** 2020-12-13

**Authors:** Seungwoo Kim, Suk Pyo Shin, Seul Ki Kim, Young Lim Ham, Han Seok Choi, Myong Jo Kim, Sang Hak Han, Ki Tae Suk

**Affiliations:** ^1^ Institute for Liver and Digestive Diseases Hallym University College of Medicine Chuncheon South Korea; ^2^ Department of Nursing Daewon University College Jecheon South Korea; ^3^ Department of Agricultural and Fisheries Precessing Korea National College of Agriculture and Fisheries Jeonju South Korea; ^4^ Division of Bioresource Sciences College of Agriculture and Life Sciences Kangwon National University Chuncheon South Korea; ^5^ Department of Pathology Hallym University College of Medicine Chuncheon South Korea

**Keywords:** eradication, gastritis, *Helicobacter pylori*, *Rhus verniciflua*

## Abstract

An antibacterial effect of fermented‐*Rhus verniciflua* extract (FRVE), an urushiol‐free extract fermented by *Fomitella fraxinea*, on *Helicobacter pylori* was evaluated in mice. Minimal inhibitory concentration of FRVE against *H. pylori* eradication was checked with serial dilution method in vitro. *H. pylori* infection‐induced mice were utilized to determine the effect of oral administration of FRVE with/without standard triple therapy (STT: metronidazole, omeprazole, and clarithromycin) on *H. pylori* colonization and gastric inflammation. *H. pylori* was clearly eradicated by FRVE at a concentration of ≥2 mg/ml in vitro. In animal study, FRVE at a concentration of ≥6 mg/ml significantly reduced colonized *H. pylori* grading (0.2 vs. 2.2, *p* < .01) and improved gastric inflammation (0.4 vs. 1.6, *p* < .01) compared to control. STT with FRVE (3 mg/ml) exerted synergistic effect on both *H. pylori* colonization grade (STT, 0.6 ± 0.9; FRVE, 1.4 ± 0.5; STT + FRVE, 0.8 ± 0.4) and gastric inflammation (STT, 0.4 ± 0.5; FRVE, 1.4 ± 0.5; STT + FRVE,1.0 ± 0.1) compared with single therapy (*p* < .01). *H. pylori* eradication rate of FRVE (6 mg/ml) was higher than that of STT (60% vs. 20%). FRVE has potential antibacterial activity against *H. pylori* infection and can be used as an additional therapy on STT.

## Graphical abstract

FRVE exhibited more potent activity against *H. pylori* compared to the standard triple therapy comprising two antibiotic agents and a proton pump inhibitor. FRVE significantly reduced *H. pylori*‐induced gastritis, although such effect was not due to altered IL‐1β or TNF‐α expression in mice.

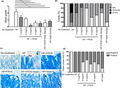

## INTRODUCTION

*Helicobacter pylori*, a gram‐negative spiral bacillus, is one of the most common bacterial pathogens worldwide (Suarez et al., 2006). *H. pylori* infection is associated with a range of gastrointestinal disorders, ranging from gastritis to gastric cancer (Ermis & Senocak Tasci, 2015). Standard triple therapy (STT) of clarithromycin, a proton pump inhibitor, and either metronidazole or amoxicillin for 1–2 weeks is used worldwide for treating *H. pylori* infection. It has a high eradication success rate of >80% (Gong et al., 2014). However, STT is associated with a number of side effects, including antibiotic resistance and relapse (Ermis & Senocak Tasci, 2015). Therefore, alternative agent with less serious side effects needs to be identified for the eradication of *H. pylori* and treatment of gastric disease.

*Rhus verniciflua* Stokes (*R. verniciflua*) is commonly known as the lacquer tree (Lee et al., 2015). *R. verniciflua* is traditionally used in oriental medicine for treating a variety of diseases. The plant is also consumed as an ingredient in sumac chicken and duck in Korea (Jeong et al., 2015). Urushiol is a major component in the sap of *R. verniciflua*. It has anti‐inflammatory, antimicrobial, and antioxidant effects in mice (Bang et al., 2014). However, clinical trials of urushiol are hampered by the fact that it is a causative agent of allergic contact dermatitis (Lee et al., 2015).

Fermented‐*Rhus verniciflua* extract (FRVE) is an urushiol‐free extract fermented by *Fomitella fraxinea* (Choi et al., 2007). In a recent study, three phenolic acids (gallic acid, protocatechuic acid, and 4‐hydroxy benzoic acid) and four flavonoids (fustin, fisetin, sulfuretin, and butein) have been detected in FRVE, with gallic acid and fisetin being its major compounds (Choi et al., 2012). FRVE also has anticancer, antioxidant, and anti‐inflammatory effects. It can protect mice against hepatosteatosis (Choi et al., 2012). Antibacterial effect of FRVE is reported in recent studies. FRVE has growth inhibitory effect on *H. pylori* (Choi et al., 2016 *)*. And a study of antibacterial activity of major constituents which are methyl gallate, fustin, and quercitrin showed antibacterial effect of fustin and methyl gallate (Jang et al., 2016). A recent study has reported that fisetin has antibacterial effect (Leotoing et al., 2013). Moreover, gallic acid has antioxidant, antiproliferative, and antitumorigenic activities (Daglia et al., 2014). However, few have been reported about its effect on *H. pylori* has not been reported yet.

The objective of the present study was to determine the minimal inhibitory concentration of urushiol‐free FRVE for *H. pylori* eradication. Reduction of *H. pylori* and improvement of gastritis after oral administration of FRVE were also compared to those by STT in C57BL/6 mice.

## MATERIALS AND METHODS

### Fermented *R. verniciflua* extracts

FRVE, a fermented extract of bark part of *R. verniciflua*, and its constituent compounds (Table 1) were provided by HS Choi at Rural Development Administration, Republic of Korea. FRVE comprises abundant fustin, fisetin, and gallic acid in MS. The method for preparing FRVE has been described by Choi previously (Choi et al., 2012). To absorb moisture in the lacquer bark, water was immersed for 1 day and drained for 1 day. Take out the lacquer bark, transfer it to a 5 L mushroom cultivation bag, put on a filter, and sterilize for 12 to 100 min. The prepared *F. fraxinea* mycelium was inoculated, cultured for 30 days at 21℃, and then dried with hot air at 50℃ to prepare fermented lacquer. FRVE was manufactured in powder form for use in experiments. The concentration was calculated according to the solvent (distilled water).

**TABLE 1:** Components of fermented‐*Rhus verniciflua* extracts

	Components	Rhus verniciflua (mg/g)	Fermented‐Rhus verniciflua (µg/g)
Utrushiol	C15:1 ~ 3	0.6	ND
Flavonoi	Fustin	20.3	87.1
	Fisetin	3.3	6.0
	Sulfuretin	0.66	3.2
	Butein	2.2	ND
	Quercetin	ND	1.9
Phenolic acid	Gallic acid	15.7	5.4

Abbreviation: ND, not detected

### Minimal inhibitory concentrations

*H. pylori* Sydney strain 1 (SS1) was obtained from *H. pylori* Korean Type Culture Collection (HpKTCC, Department of Microbiology, Gyeongsang University College of Medicine, Jinju, Korea). *H. pylori* was maintained on Mueller–Hinton agar containing 5% sheep blood (MH‐BAP, Hangang, Gunpo, Korea) at 37°C with 10% CO_2_. Agar dilution method was used to determine minimal inhibitory concentrations (MICs). FRVE was subjected to serial dilution, yielding concentrations of 0.5 to 4 mg/ml. It was then mixed with MH‐BAP. *H. pylori* was transported to half‐plates from 72‐hr‐subcultured normal MH‐BAP plates using smear culture method. Plates were incubated at 37°C in an incubator with 10% CO_2_. After incubation for 72 hr, MICs of FRVEs were evaluated and its appropriate concentration for in vivo study was determined. Rough surfaces of half plate suggest living organisms on its concentration and smooth surface means opposition.

### Animals

**FIGURE 1:**
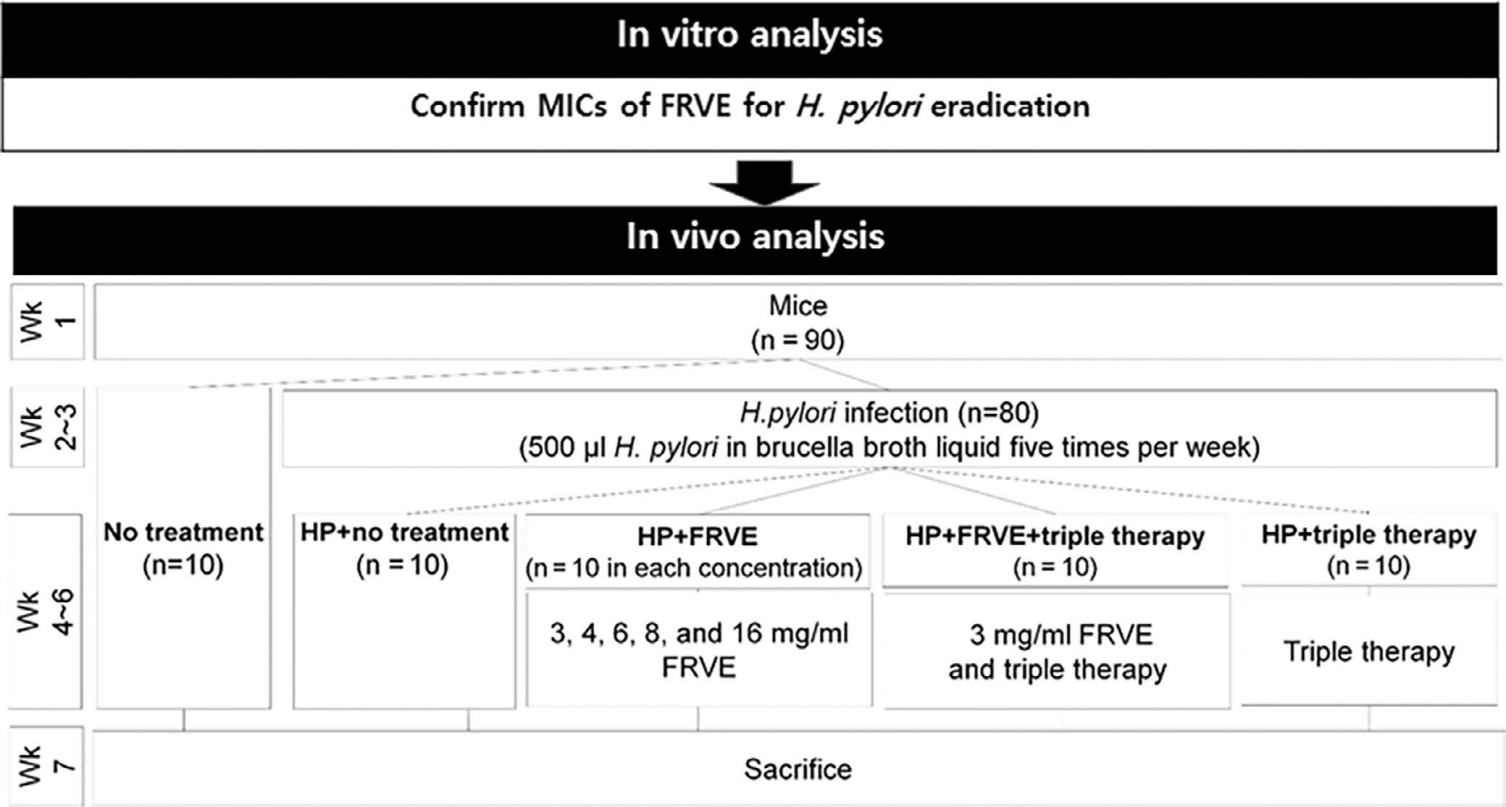
Study design and flowchart of experiments. FRVE, fermented‐*Rhus verniciflua* extract

All animals received humane care in this study. All procedures were conducted in accordance with National Institutes of Health Guidelines for the Care and Use of Laboratory Animals. All procedures were approved by Institutional Animal Care and Use Committee of Hallym University College of Medicine (2013‐131; 2014‐71; 2015‐21). Six‐week‐old specific pathogen‐free male C57BL/6 mice were purchased from Dooyeol Biotech (Seoul, Korea). All mice were housed individually in steel microisolator cages at 22 ± 2°C with 12 hr/12 hr of light/dark cycle. They were provided free access to water and food throughout the experiment period. They were monitored daily. These mice were divided into the following groups (Figure 1): no treatment (*n* = 10), HP (*H. pylori* infection, *n* = 10), HP + FRVE (*H. pylori* infection and treatment with FRVE, *n* = 50), HP + FRVE+triple (*H. pylori* infection and treatment with FRVE + standard triple therapy, *n* = 10), and HP + triple (*H. pylori* infection and treatment with standard triple therapy *n* = 10).

*H. pylori* SS1 was maintained on Brucella agar [28 g Brucella broth powder (R&D Systems, Minneapolis, MN, USA) with 15 g agar per liter] containing 10% horse serum (Thermo Fisher Scientific, Waltham, MA, USA) at 37°C in an incubator with 10% CO_2_. *H. pylori* was incubated for 72 hr and transferred to Brucella broth liquid containing 10% horse serum. Before infection, mice were fasted for 4 hr. Optical density of *H. pylori* suspension was adjusted to 10, corresponding to 6.7 × 10^9^ CFU/ml (Navabi et al., 2013). For standard triple therapy treatment, 400 mg/kg metronidazole, 20 mg/kg omeprazole, and 250 mg/kg clarithromycin were mixed in drinking water (Loughlin et al., 2003). Standard triple therapy was administered five times per week.

In the second week, mice in the four groups were infected with *H. pylori* for 2 weeks. In the fourth week, mice in the HP + FRVE group were divided into the following five subgroups and treated with 3, 4, 6, 8, or 16 mg/ml of FRVE suspension. Treatment of the FRVE suspension was performed 5 times per week for 3 weeks. Mice in the HP + FRVE+triple group were treated with 3 mg/ml of FRVE suspension for 3 weeks. In the fifth week, mice in the triple groups were treated with standard triple therapy for 2 weeks. Mice were euthanized in the seventh week.

### *H. pylori* infection and treatments

Mice in groups of HP were inoculated with 500 μl *H. pylori* in broth liquid five times per week. FRVEs were suspended in drinking water at 3, 4, 6, 8, or 16 mg/ml. Subsequently, mice in groups of HP + FRVE and HP + FRVE+triple were treated with 200 μl of FRVE suspension five times per week. *H. pylori* eradication regimen was normalized to body weight of mouse five times per week. A 19‐guage feeding needle was used to perform all inoculations and treatments. At the end of the experiment, mice were euthanized by inhalation anesthesia overdose (isoflurane, Aerane; Baxter, Deerfield, IL, USA). Whole blood and stomach were collected. Whole blood (~0.6 ml) was centrifuged (19,000 × *g*) to collect serum. Livers and small intestines were rapidly excised and stored at −80°C.

Stomachs were divided into three parts which contain corpus and antrum for rapid urease test (CLO test kit, Greencross, Yongin, Korea), pathological analysis, and determination of proinflammatory cytokine levels. One portion of stomach was fixed in 10% formalin for pathological analysis. The second portion was used to detect *H. pylori* infection using CLO test kit according to the manufacturer's instructions. The remaining portion was stored at −80°C until measurement of proinflammatory cytokine levels.

### Histology

A midline abdominal incision was performed, and the stomachs were collected for analysis. H. pylori infection was confirmed by rapid urease test kit or pathology confirmation. The stomach was incised along the greater curvature and fixed in 10% neutral buffered formalin. The tissue was embedded, cut at 5 µm, and stained with hematoxylin and eosin/giemsa staining. The degree of inflammation of the gastric mucosa was measured based on the updated Sydney system (Dixon et al., 1996) using a 4‐point scale for grading *H. pylori* and inflammation severity: grade 0, normal; grade 1, mild; grade 2, moderate; and grade 3, severe. A histologist who was blinded to experimental conditions analyzed all specimens.

### Cytokine

Stomach tissue was homogenized using a complete Bio‐Plex Cell Lysis Kit (Bio‐Rad, Hercules, CA, USA). Homogenization was performed using 100 μL of cell lysis buffer and 500 μL PBS per 100 mg tissue. Expression of proinflammatory cytokines in stomach tissue and serum was analyzed by enzyme‐linked immunosorbent assay (ELISA, Bio‐Plex Pro Mouse Cytokine Assay Kit, Bio‐Rad) according to the manufacturer's instructions.

#### Statistical analysis

Statistical analysis was performed using Prism V.5.0 (GraphPad, San Diego, CA, USA) and SPSS, V.18.0 (SPSS Inc., Chicago, IL, USA). Differences in *H. pylori* colonization grade, inflammation grade, and cytokine expression level among groups were calculated by one‐way analysis of variance (ANOVA) and Tukey's post hoc test. A *P* value of less than 0.05 was considered statistically significant.

## RESULTS

### Minimal inhibitory concentrations of FRVEs and *H. pylori* eradication

**FIGURE 2:**
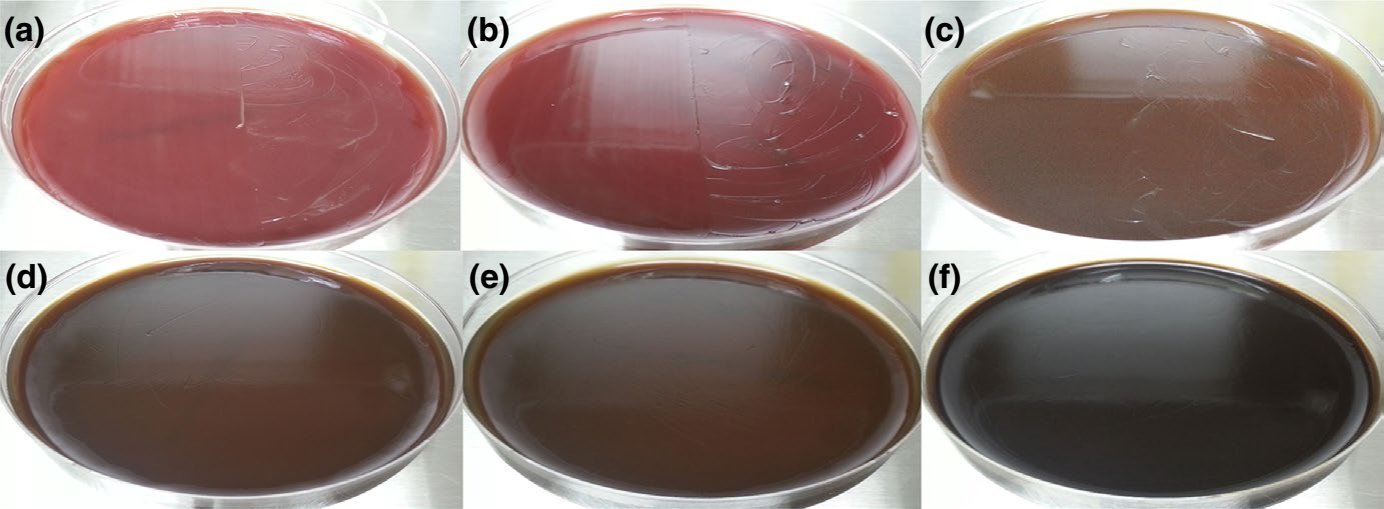
MIC of FRVEs in vitro. H. pylori was cultured in half‐right side of MH‐BAP with (a) 0 mg/ml FRVE; (b) 1 mg/ml FRVE; (c) 1.5 mg/ml FRVE; (d) 2 mg/ml FRVE; (e) 3 mg/ml FRVE; and (f) 4 mg/ml FRVE. *H. pylori* proliferation was unaffected by 1 or 1.5 mg/ml of FRVE. FRVE, fermented‐Rhus verniciflua extract

FRVE at 2 to 4 mg/ml eradicated *H. pylori*. However, *H. pylori* proliferation was unaffected by 1 or 1.5 mg/ml of FRVE (Figure 2). Treatment with 2 mg/ml of FRVE reduced *H. pylori* grade in mice (data not shown). Thus, treatment with a high concentration of FRVE might eradicate *H. pylori*. To test this possibility, mice were treated with 3, 4, 6, 8, or 16 mg/ml of FRVE for 3 weeks.

**FIGURE 3:**
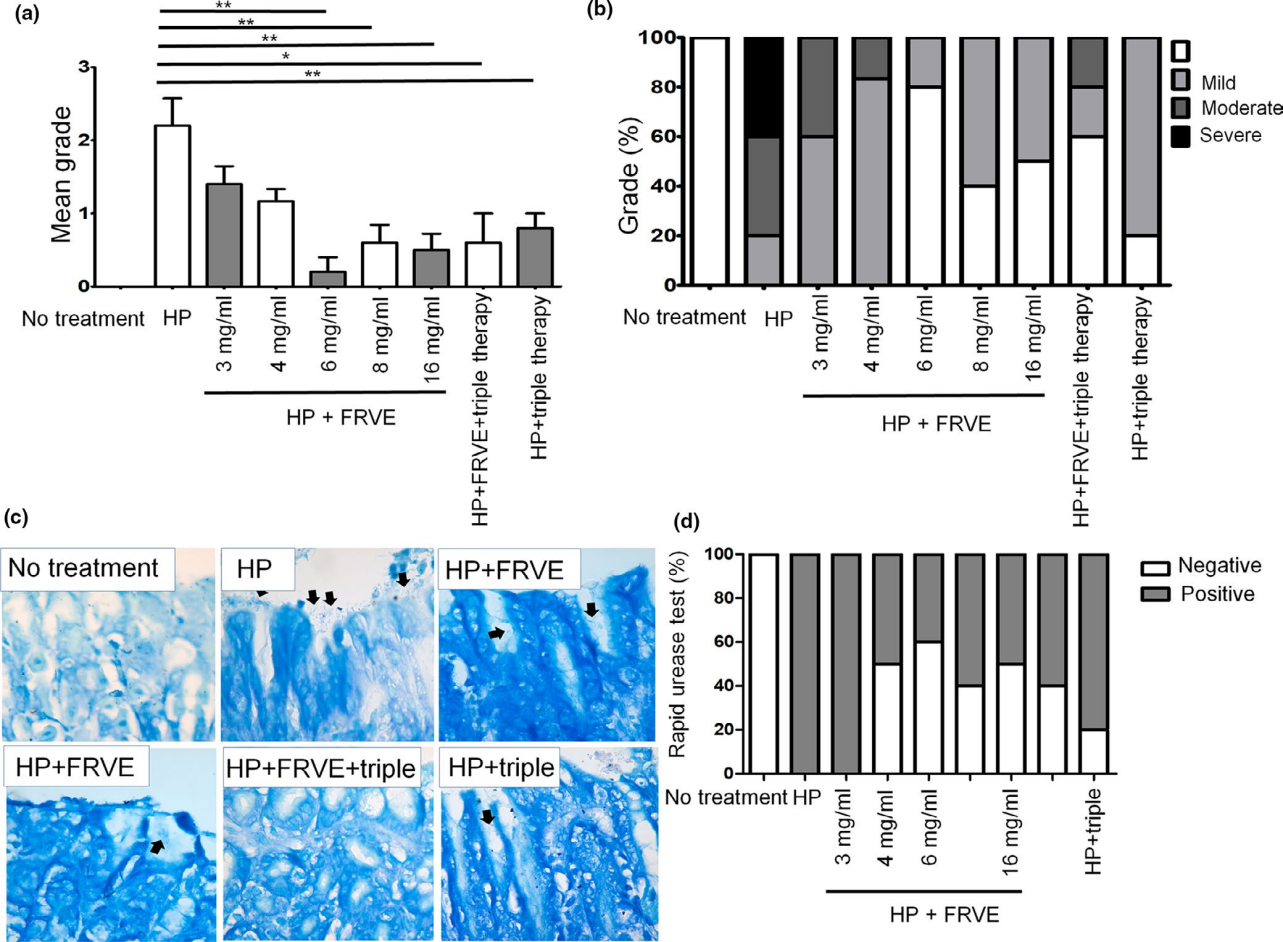
*H. pylori* colonization of histological analysis in mice treated with FRVE after *H. pylori* infection during 2 weeks. Mice were infected *H. pylori* for 2 weeks and treated with FRVE. (a) Mean grade of antrum and corpus, (b) percentage of grade on antrum, and (c) representative images of colonized *H. pylori* of Giemsa staining in stomach of mice. (d) Percentage of response in rapid urease test. In HP, HP + FRVE, and HP + triple therapy group, *H. pylori* are located in the mucus of outer mucosal layer (arrow). *p < .05; **p < .01. FRVE, fermented‐*Rhus verniciflua* extract

In the analysis of antrum and corpus, *H. pylori* colonization in HP group had a mean grade of 2.2 ± 0.8 (20% mild, 40% moderate, and 40% severe)(Figure 3). *H. pylori* colonization in HP + FRVE 3 mg/ml subgroup had a mean grade of 1.4 ± 0.5. *H. pylori* colonization in subgroup of HP + FRVE 4 mg/ml had a mean grade of 1.2 ± 0.4. *H. pylori* colonization in subgroups of HP + FRVE 3 mg/ml and HP + FRVE 4 mg/ml tended to be less severe than that in HP group. *H. pylori* colonization in subgroup of HP + FRVE 6 mg/ml had a mean grade of 0.2 ± 0.4. *H. pylori* colonization in subgroup of HP + FRVE 8 mg/ml had a mean grade of 0.6 ± 0.5. *H. pylori* colonization in subgroup of HP + FRVE 16 mg/ml had a mean grade of 0.5 ± 0.5. The subgroups of HP + FRVE 6 mg/ml, HP + FRVE 8 mg/ml, and HP + FRVE 16 mg/ml showed significantly (*p* < .01) lower *H. pylori* colonization than that in HP group. *H. pylori* colonization in HP + FRVE+triple group had a mean grade of 0.6 ± 0.9. *H. pylori* colonization in HP + triple group had a mean grade of 0.8 ± 0.4. *H. pylori* colonization in both HP + FRVE+triple and HP + triple group showed significantly lower grade compared to that in HP group (*p* < .05).

Rapid urease test revealed that eradication rates of *H. pylori* by FRVE was dose‐dependent (Figure 3). HP and HP + FRVE 3 mg/ml showed 100% of positive reaction for gastric *H. pylori*. However, HP + FRVE 4 mg/ml and HP + FRVE 6 mg/ml showed 50% and 60% negative reaction for gastric *H. pylori*, respectively. HP + FRVE 8 mg/ml and HP + FRVE 16 mg/ml had 40% and 50% negative reaction for gastric *H. pylori*, respectively. HP + FRVE+triple and HP + triple group showed 40% and 20% negative reaction for gastric *H. pylori*, respectively. These results were similar to those of histological analysis. Especially, 6 mg/ml FRVE effectively reduced *H. pylori* grade and increased *H. pylori* eradication rate. Moreover, HP + FRVE+triple showed superior eradication rate than HP + FRVE 3 mg/ml or HP + triple therapy. These results were similar to those of *H. pylori* grade based on histological analysis.

### Improvement in gastritis

**FIGURE 4:**
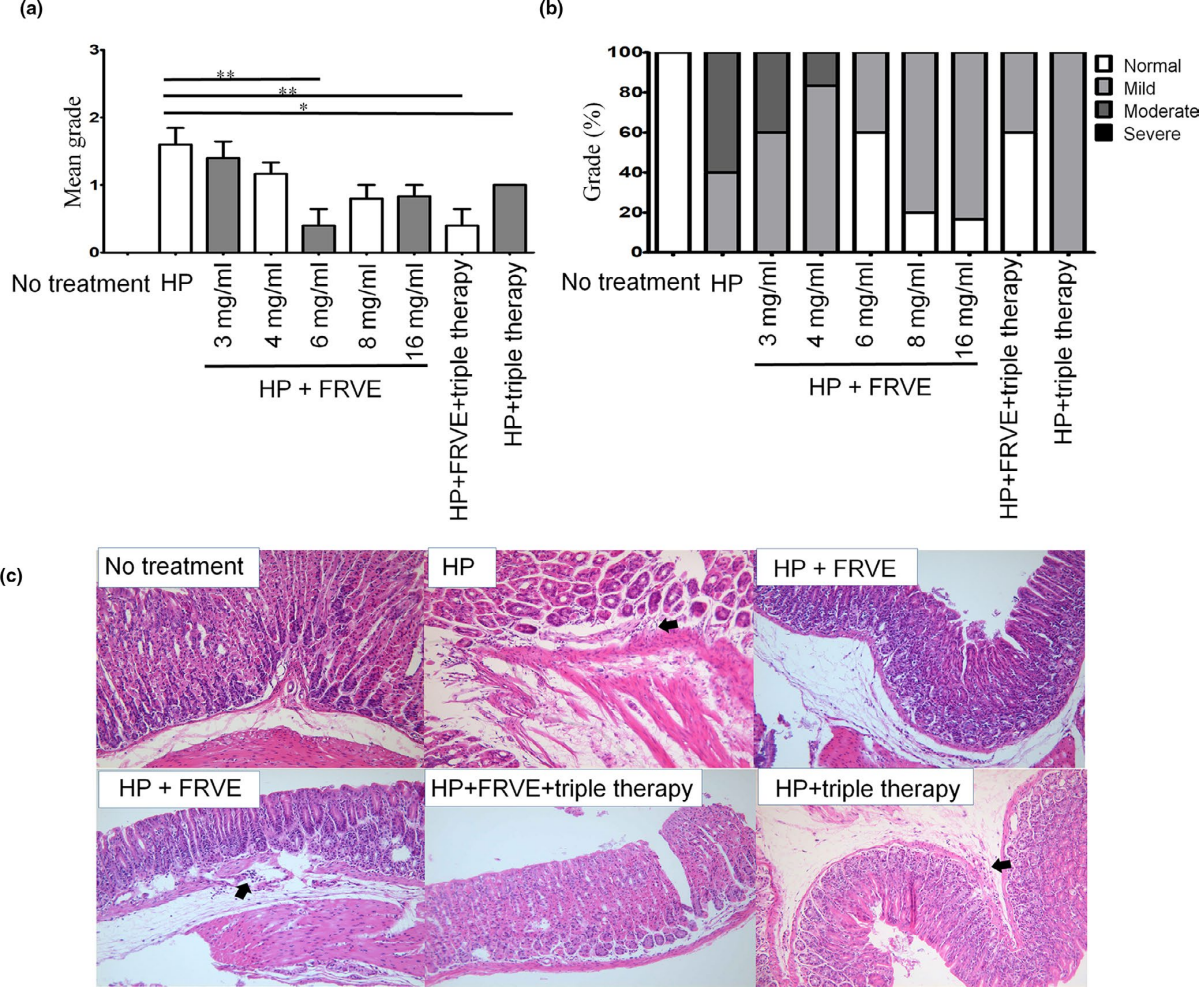
Gastric inflammation of histological analysis in mice treated FRVE after *H. pylori* infection. Mice were infected *H. pylori* for 3 weeks and treated the FRVE. (a) Mean grade of antrum and corpus, (b) percentage of grade on antrum, and (c) representative images of inflammation in stomach of mice. In HP, HP + FRVE, and HP + triple therapy group, neutrophil and mononuclear cells are infiltrated in the stomach wall (black arrow). Significances are shown above bars; *p < .05; **p < .01

Regarding inflammation improvement, inflammation in HP had a mean grade of 1.6 ± 0.5 (Figure 4). This finding was shown on antrum analysis not corpus. Inflammation in subgroup of HP + FRVE 3 mg/ml had a mean grade of 1.4 ± 0.5. Inflammation in subgroup of HP + FRVE 4 mg/ml had a mean grade of 1.2 ± 0.4. Inflammation grade in subgroup of HP + FRVE 3 mg/ml or HP + FRVE 4 mg/ml tended to be lower than that in HP group. Inflammation in subgroup of HP + FRVE 6 mg/ml had a mean grade of 0.4 ± 0.5, which was significantly (*p* < .01) lower than in HP group. Inflammation in subgroup of HP + FRVE 8 mg/ml had a mean grade of 0.8 ± 0.4. Inflammation in subgroup of HP + FRVE 16 mg/ml also had a mean grade 0.8 ± 0.4. Inflammation grades in HP + FRVE 8 mg/ml and HP + FRVE 16 mg/ml subgroups tended to be lower than that in HP group. However, they were higher than that in HP + FRVE 6 mg/ml subgroup. Inflammation grade in HP + triple group was 1.0 (100% mild), similar to that in HP + FRVE 8 mg/ml and HP + FRVE 16 mg/ml subgroups. However, it was significantly (*p* < .05) lower than that in HP group. Inflammation in HP + FRVE+triple group had a mean grade of 0.4 ± 0.5, similar to that in HP + FRVE 6 mg/ml subgroup. However, it was significantly (*p* < .01) lower than that in HP group. No gastric inflammation was detected in the control group without any treatment.

### Inflammatory cytokine

**FIGURE 5:**
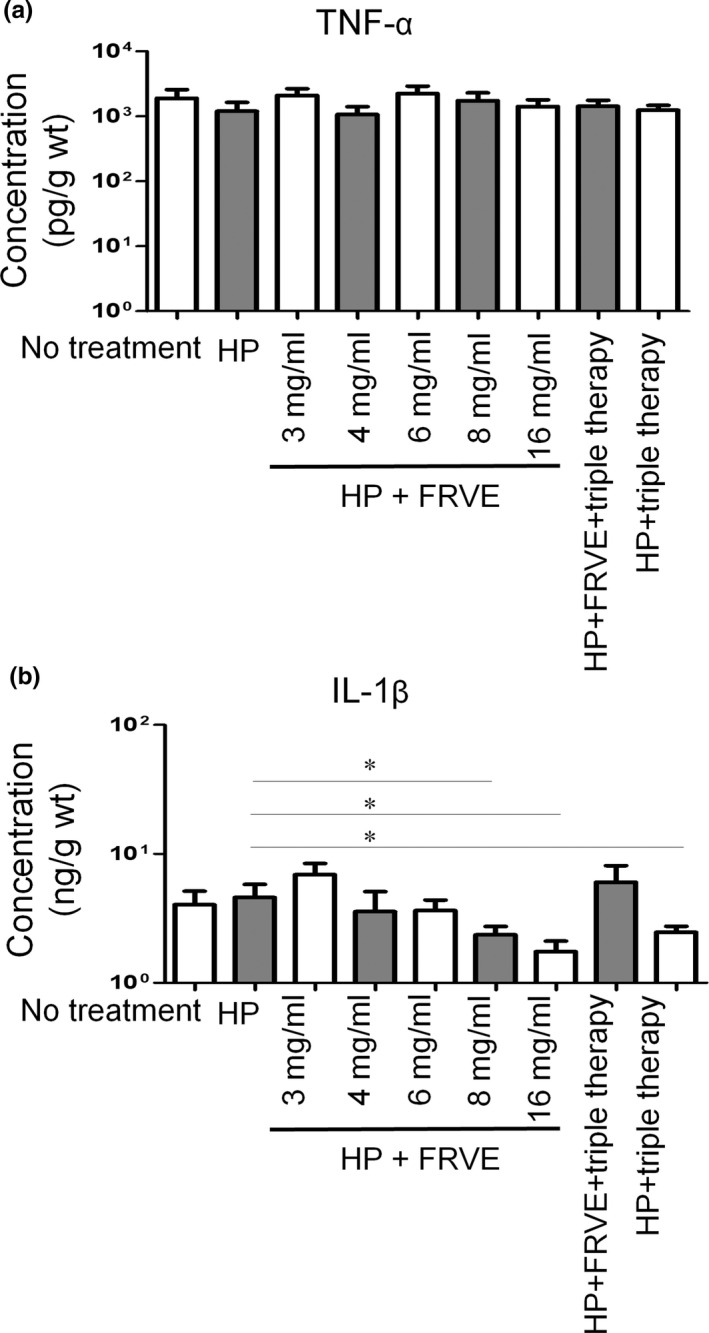
Figure 1. Proinflammatory cytokines level in stomach. (a) Expression of TNF‐α in stomach of mice treated FRVE after *H. pylori* infection for 3 weeks (b) Expression of IL‐1β in stomach of mice treated FRVE after *H. pylori* infection for 3 weeks

After *H. pylori* infection for 2 weeks, TNF‐α concentrations did not differ significantly among groups (Figure 5). However, IL‐1β concentration in subgroup of HP + FRVE 8 and 16 mg/ml and HP + triple group was significantly lower than that in HP group.

## DISCUSSION

In this study, the MIC of FRVE against *H. pylori* in vitro and the concentration that resulted in the greatest reduction in *H. pylori* grades and improvement of inflammation in vivo were investigated. Application of 2 mg/ml of FRVE was highly effective in vitro while 6 mg/ml of FRVE was highly effective animal study. In this study, FRVE contained higher level of fustin than other components (Table 1). Previous reports have demonstrated that fustin possesses cell proliferation‐inhibiting and antiviral activities (Kang et al., 2012). Results for this study revealed that fustin also had antibacterial effect against *H. pylori* and anti‐inflammatory activity. In our results, fustin revealed main component in the FRVE. In a previous report, fustin exerts neuroprotection from cell death and fustin flavonoid attenuates beta‐amyloid (1–42)‐induced learning impairment (Jin et al., 2009; Park et al., 2007). Fisetin alleviated kidney inflammation and apoptosis to protect against LPS‐induced septic condition (Ren et al., 2020) and reduced inflammation on toxicity (Hytti et al., 2017). Gallic acid also has beneficial effects on various diseases including ulcer (Akbari, 2020; Zhou et al., 2020). Taken together, these findings suggest that FRVE has anti‐*H. pylori* activity and consequently may be used as a supplementary therapy in *H. pylori*‐associated stomach disease. Further studies about effects of each component are needed in the future.

Histological analysis by H&E and Giemsa staining with urease test using CLO test kit were used to detect *H. pylori* in this study. Interestingly, rapid urease test revealed that *H. pylori* eradication rate was increased by 6 mg/ml FRVE. FRVE at 6 mg/ml shown higher eradication rate (60%) compared to 0% in the control group. This rate (60%) was also higher than that (20%) of the standard triple therapy. This results suggest that FRVE may be used as a supplementary agent or single agent in anti‐*H. pylori* regimen, but further research is required for the clinical application.

A reduction in *H. pylori* grades according to the upgraded Sydney system can protect against *H. pylori*‐induced gastritis and gastric cancer (Schenk et al., 2000). Our results showed that *H. pylori* colonization grade in HP was decreased to 0.2 ± 0.4 after treatment with 6 mg/ml of FRVE. In addition, inflammation grade was decreased to 0.4 ± 0.5. These results suggest that *H. pylori* caused gastric inflammation can be ameliorated by FRVE. Therefore, FRVE might have the potential as a therapeutic agent for *H. pylori*‐induced gastritis and gastric cancer.

Our results showed that application FRVE (a concentration of 6 or 3 mg/ml) together with standard triple therapy reduced *H. pylori* grades and improved inflammation. The standard triple therapy with proton pump inhibitor and antibiotics (amoxicillin, metronidazole, or clarithromycin) has been used to eradicate *H. pylori* for a long time, and resistance of *H. pylori* to these antibiotics has increased (Testerman & Morris, 2014).

In this study, FRVE at 2 mg/ml eradicated *H. pylori* in vitro. This concentration is lower than those of antibiotics or proton pump inhibitors used in the standard triple therapy, in which higher doses of amoxicillin, metronidazole, and clarithromycin are used. Because the standard triple therapy has been used to eradicate *H. pylori* for a long time, resistance of *H. pylori* to these antibiotics has increased (Testerman & Morris, 2014). Our results showed that application FRVE at 6 or 3 mg/ml together with standard triple therapy reduced *H. pylori* grades and improved inflammation.

In the present work and in prior studies that used urushiol‐free FRVE as a single treatment or repeated for 90 days, no toxicity was recorded (Shin et al., 2013). Safety evaluation conducted by the Rural Development Administration proved reliable safety in toxicity test, antigen test, repeated DRF skin test, and genetic toxicity test using rats and beagle dogs. Only vomiting that was not result from systemic toxicity was reported in a beagle dog with 2,000 mg/kg of urushiol‐free FRVE. Therefore, FRVE is likely to be safe for use as an antibacterial agent. It has been reported that FRVE has anticancer, antigrowth, anti‐inflammatory, antioxidant, antiviral, and neuroprotective effects in vitro (Choi et al., 2014; Sapkota et al., 2010). Therefore, FRVE has been subjected to clinical trials for various diseases (Lee et al., 2011).

Green tea, muscadine grape skin, broccoli, and cranberry have been investigated in vitro and/or in vivo as potential treatments for *H. pylori* (Brown et al., 2011). However, these studies did not make use of animal models and their concentrations were higher than 2 mg/ml, the concentration of FRVE used in this work. Thus, FRVE could be used as anti‐*H. pylori* agent at a low concentration for a short period.

Gastric IL‐1β expression is associated with *H. pylori* infection while urushiol therapy can reduce its expression (Lee et al., 2010; Suk et al., 2011). In our study, IL‐1β level follows previous reports. However, TNF‐α expression levels were not affected by FRVE. This might be due to the low concentration of sulfuretin present in FRVE. This suggests that FRVE might have exerted its beneficial effects through its direct cytotoxic effect on *H. pylori* other than through expression of TNF‐α. Further study should focus on identifying other cytokines associated with *H. pylori* infection and FRVE therapy.

**FIGURE 6:**
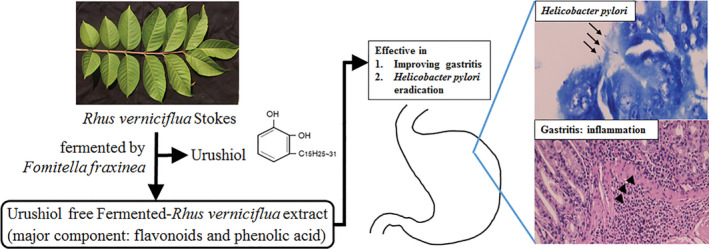
Study summary

FRVE exhibited more potent activity against *H. pylori* compared to the standard triple therapy comprising two antibiotic agents and a proton pump inhibitor (Figure 3). In addition, FRVE significantly reduced *H. pylori*‐induced gastritis, although such effect was not due to altered IL‐1β or TNF‐α expression in mice. Furthermore, the standard triple therapy together with a low concentration of FRVE exerted synergistic effect. Therefore, FRVE might be used as a potential adjuvant therapeutic agent for treating *H. pylori*‐induced gastric disease (Figure 6).
